# KIAA1114, a full-length protein encoded by the trophinin gene, is a novel surface marker for isolating tumor-initiating cells of multiple hepatocellular carcinoma subtypes

**DOI:** 10.18632/oncotarget.1677

**Published:** 2014-03-31

**Authors:** Sae Won Kim, Hyun Gul Yang, Moon Cheol Kang, Seungwon Lee, Hong Namkoong, Seung-Woo Lee, Young Chul Sung

**Affiliations:** ^1^ Department of Life Sciences, Pohang University of Science and Technology (POSTECH), Pohang, Gyungbuk, Republic of Korea; ^2^ Integrative Biosciences & Biotechnology, Pohang University of Science and Technology (POSTECH), Pohang, Gyungbuk, Republic of Korea

**Keywords:** Tumor-initiating cells, hepatocellular carcinoma, KIAA1114, cell surface marker, monoclonal antibody

## Abstract

Identification of novel biomarkers for tumor-initiating cells (TICs) is of critical importance for developing diagnostic and therapeutic strategies against cancers. Here we identified the role of KIAA1114, a full-length translational product of the *trophinin* gene, as a distinctive marker for TICs in human liver cancer by developing a DNA vaccine-induced monoclonal antibody targeting the putative extracellular domain of KIAA1114. Compared with other established markers of liver TICs, KIAA1114 was unique in that its expression was detected in both alpha fetoprotein (AFP)-positive and AFP-negative hepatocellular carcinoma (HCC) cell lines with the expression levels of KIAA1114 being positively correlated to their tumorigenic potentials. Notably, KIAA1114 expression was strongly detected in primary hepatic tumor, but neither in the adjacent non-tumorous tissue from the same patient nor normal liver tissue. KIAA1114^high^ cells isolated from HCC cell lines displayed TIC-like features with superior functional and phenotypic traits compared to their KIAA1114^low^ counterparts, including tumorigenic abilities in xenotransplantation model, in vitro colony- and spheroid-forming capabilities, expression of stemness-associated genes, and migratory capacity. Our findings not only address the value of a novel antigen, KIAA1114, as a potential diagnostic factor of human liver cancer, but also as an independent biomarker for identifying TIC populations that could be broadly applied to the heterogeneous HCC subtypes.

## INTRODUCTION

Durable complete remission has been rarely achieved with conventional anticancer strategies owing to sustained survival or continuous reappearance of therapy-resistant tumor cells that cause locoregional relapse and distant metastasis. Such observation led to the emergence of “cancer stem cell” hypothesis that tumors, like normal somatic tissues, contain distinct stem cell-like populations having strong regeneration capacity and drug resistance. The exact definition of “cancer stem cells (CSCs)” was recently provided by Valent et al. that CSCs are a subset of neoplastic stem cells that drives indefinite proliferation of malignant clones and production of an apparent cancer [1]. They also suggested that the term “tumor-initiating cells (TICs)”, which has been widely used to describe experimentally identified CSCs, may be applied to characterize cells that are capable of initiating the formation of a detectable, continuously growing tumor in xenograft models [1]. CSCs are thought to stand at the pinnacle of tumor hierarchy and be capable of producing differentiated progenies to recapitulate the clonal heterogeneity of parental tumors [2]. Thus, the resistance of tumors to existing therapies as well as their recurrence and metastatic spread are possibly due to the existence of CSCs bearing indefinite self-renewal capacities. CSCs tend to express elevated levels of ATP-binding cassette (ABC) transporters, aldehyde dehydrogenase 1, pro-survival and DNA repair proteins, all of which contribute to their intrinsic drug resistance [3]. In addition, a number of studies on the metastasis of solid tumors have shown that epithelial-mesenchymal transition (EMT) triggers cells located at the invasive edge of the primary tumor to obtain stemness properties along with motility and invasiveness, and these migrating CSCs are endowed with the unique abilities to colonize and initiate tumor growth at the secondary site [4, 5]. Considerable efforts have been made over the last few decades to identify surface markers that are exclusively expressed on CSCs, as developing therapeutics targeting CSCs will undoubtedly open new ways for the treatment of human cancers. Despite the comprehensive list of CSC markers, however, clinical transition of most available markers has been rarely achieved since the application of a sole marker for broad range of tumor subtypes has been frequently hampered by intertumoral and intratumoral heterogeneity of a given cancer.

In human hepatocellular carcinoma (HCC), a number of TIC markers have been identified including CD133, EpCAM, and CD90, whose functions were well-characterized using both cell lines and primary tumor specimens [6]. Importantly, however, none of these markers were universally expressed within diverse subtypes of liver cancer. In HCC cell lines, which can be classified by alpha fetoprotein (AFP) status [7], expressions of CD133 and EpCAM were only detected in AFP^+^ cells, whereas CD90 expression was exclusively found in AFP^−^ counterparts [8]. Furthermore, in primary HCC, not only did the level of EpCAM and CD90 expression vary greatly among different individuals, but the cell populations expressing EpCAM and CD90 were distinctively localized without overlap even in a given tumor derived from a single patient [9]. These findings provide clear evidence for the existence of heterogeneity in primary liver cancer and the differential expression pattern of TIC markers depending on HCC subtypes. Therefore, further identification of biomarkers that could be broadly used to identify TICs from multiple tumor subtypes may bring significant clinical benefits in terms of diagnosis and therapy.

MAGE (Melanoma-associated antigen) superfamily proteins have been considered as attractive therapeutic targets owing to their specific expression in cancer. *MAGE-D* genes are recently discovered *MAGE* subfamily with distinguished features in terms of their genomic structures and expression patterns. Among the members of *MAGE-D* family, *trophinin* (also known as *MAGE-D3*) is particularly unique in that it can encode multiple translational products having pleiotropic functions in various biological processes. Previous reports have suggested that the presence of numerous translational initiation sites in *trophinin* mRNA and utilization of different alternative splicing could generate three major types of proteins – KIAA1114, TROPHININ, and MAGPHININs [10, 11]. Although a number of studies have been performed on dissecting the physiological role and function of TROPHININ in embryo implantation and cancer progression [12], only few studies have been conducted on a full-length protein encoded by the *trophinin* gene known as KIAA1114, whose cDNA coding sequence was initially identified from human brain [13]. Despite the reported expression of *KIAA1114* at the transcriptional level, physiological evidence for the existence of KIAA1114 at the protein level has never been reported to date, making it a “hypothetical” protein for more than a decade.

In this study, we provide the first direct evidence for the presence of KIAA1114 at the protein level in cancer cells by utilizing a monoclonal antibody (mAb) raised against the extracellular domain of KIAA1114 antigen and propose its potential role as a prognostic factor, and more significantly, as a distinctive and versatile TIC surface marker for multiple subtypes of human liver cancer.

## RESULTS

### KIAA1114, the full-length translation product of the *trophinin* gene, is a transmembrane protein localized on the cell surface

**Figure 1:**
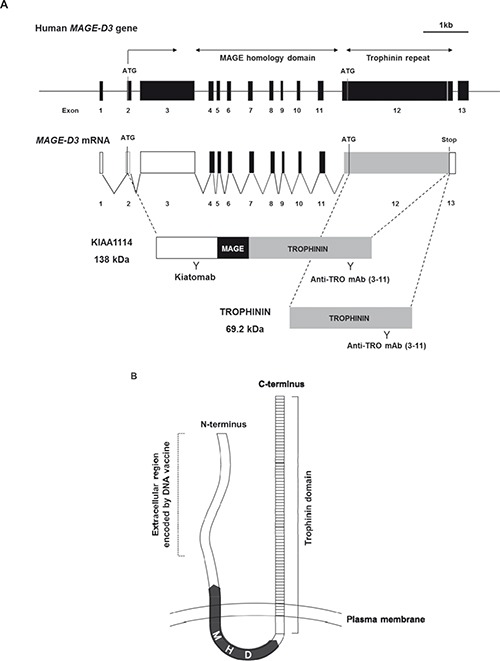

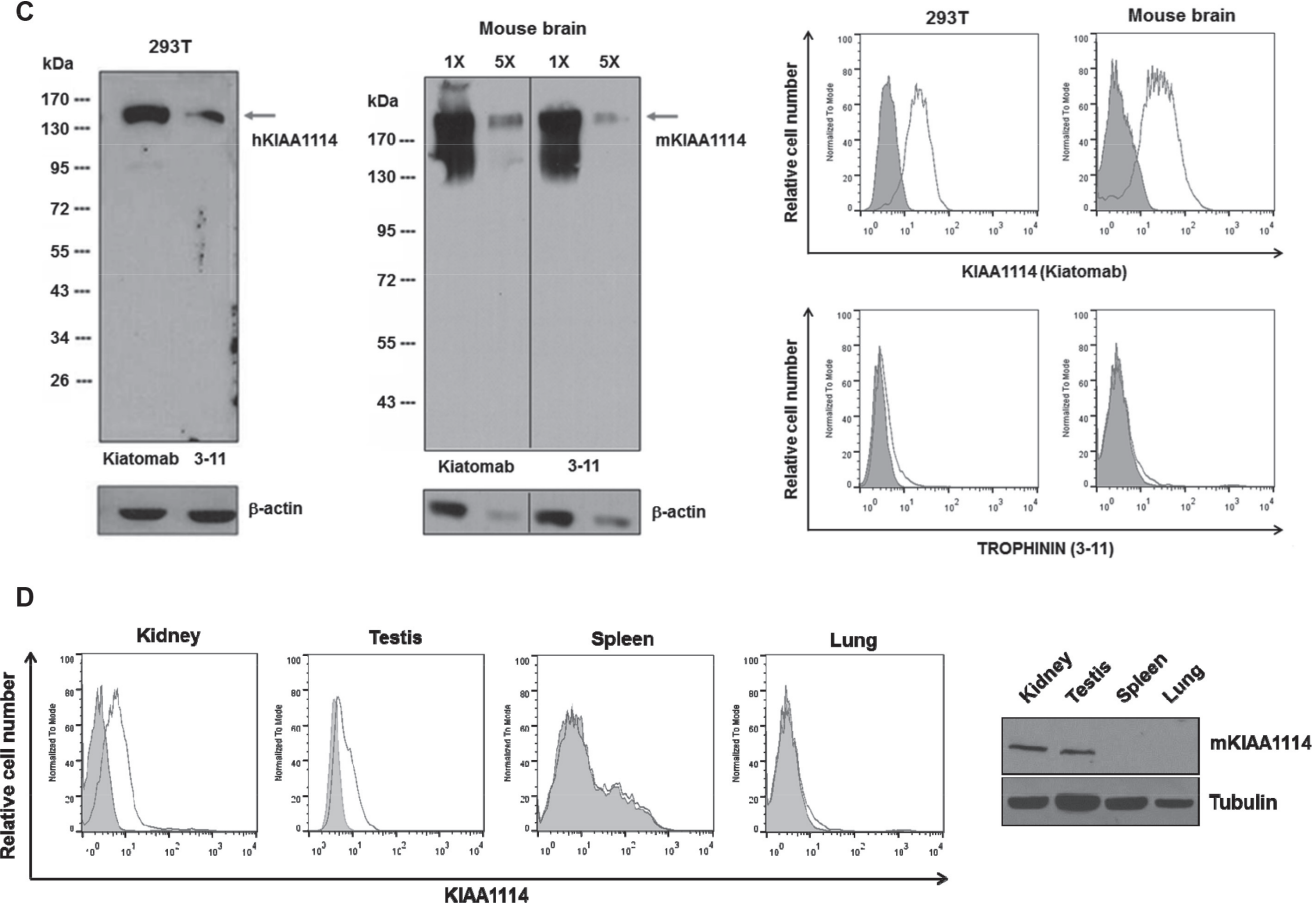
Identification and localization of KIAA1114 with a novel anti-KIAA1114 mAb, Kiatomab **(A)** Schematic structures of human MAGE-D3 gene, transcript, and protein products – KIAA1114 and TROPHININ (MAGPHININ proteins are not shown). Regions recognized by Kiatomab and anti-TROPHININ mAb (3–11) are indicated. **(B)** Schematic diagram showing putative transmembrane topology of KIAA1114 protein. Predicted locations of TROPHININ domain, MAGE homology domain (MHD), and partial N-terminal domain encoded by DNA vaccine used for Kiatomab generation are indicated. **(C)** Western blot and flow cytometric analysis for recognition of human and mouse KIAA1114 by Kiatomab and 3–11 mAb using 293T cells and mouse brain tissue. β-actin was used as a loading control for Western blot. In flow cytometry, KIAA1114 expression is indicated as open histogram and isotype controls are represented as shaded histogram. **(D)** Analysis of KIAA1114 expression in various murine tissues by flow cytometry and Western blot. Isotype control and Kiatomab stainings are indicated as describe above for flow cytometry. Tubulin was used as an internal control for Western blot assay.

The nomenclature for a full-length protein encoded by the *trophinin* gene has not been well-defined [11, 14], as experimental evidence for the expression of KIAA1114 in vitro or in vivo has never been provided by earlier studies. In the present study, we used the term “KIAA1114” to describe the full-length product translated from *trophinin* mRNA (Figure 1A). Hydropathy analysis using the topology prediction program TMpred [15] revealed that KIAA1114 is a transmembrane protein with the N-terminus outside the cell (Supplementary Figure 1). Moreover, as previously suggested [16], KIAA1114 is predicted to contain an intracellular MAGE-homology domain and a trophinin domain that traverses the plasma membrane. Although hydropathy analyses performed in the present and previous studies proposed that a trophinin domain spans the lipid bilayer multiple times [17], a recent review raised a possibility that TROPHININ is a single-pass, type II transmembrane protein that utilizes the majority of its extracellular decapeptide repeats for homophilic adhesion [18]. Accordingly, we proposed that KIAA1114 is a double-pass, type III transmembrane protein with N- and C-termini facing the outside of the cell (Figure 1B). Although thorough structural analysis is required to determine exact location of transmembrane regions, TMPred suggested that the first membrane-spanning segment lies within a MAGE-homology domain and the second one locates near the N-terminus of a trophinin domain (Supplementary Figure 1).

To develop a novel mAb targeting KIAA11114, mice were immunized with the gene encoding partial N-terminal extracellular domain of human MAGE-D3 (Figure 1B). DNA immunization was selected over conventional peptide/protein immunization, as it allows spontaneous formation of native folded proteins in vivo and subsequent generation of high-avidity antibodies recognizing antigens with proper conformation [19, 20]. To investigate whether the resulting mAb, named Kiatomab, could recognize human KIAA1114 protein, Western blot analysis was performed using human kidney 293T cell lysates, as the expression of *magphinin*/*trophinin* transcript in renal tissues has been previously suggested [21]. As a result, we found that Kiatomab reacted with a full-length, 138 kDa form of human KIAA1114. Nextly, to examine whether Kiatomab has cross-reactivity to mouse KIAA1114, further analysis was carried out on extracted proteins from mouse brain, which is known to express high level of *trophinin* transcripts [16]. Immunoblot analysis with Kiatomab revealed a single band corresponding to 196 kDa mouse KIAA1114. The expression of human and mouse KIAA1114 in the same lysates were also identified by the commercial anti-TROPHININ mAb (clone 3–11) which was used as a positive control (Figure 1C, left panel). Moreover, the reactivity of Kiatomab to 293T and primary mouse brain cells was further confirmed by surface staining analysis, implicating the cell surface localization of KIAA1114. In contrast, 3–11 mAb failed to show noticeable reactivity in equivalent experimental conditions (Figure 1C, right panel). These findings differ from a prior study that detected cell surface expression of TROPHININ on stable transfectants of a human testicular seminoma cell line overexpressing TROPHININ using 3–11 mAb by flow cytometry [22]. The exact causes of these differences remain unclear, but the disparities in the nature of cell lines used (normal untransfected cells versus stably transfected cells) and subsequent discrepancies in the copy number of the genes encoding KIAA1114 or TROPHININ may partially be responsible for yielding distinct results.

To assess whether cell surface expression of KIAA1114 correlates with reported expression pattern of *trophinin* mRNA, KIAA1114 expression in primary cells derived from various murine tissues were analyzed by flow cytometry. Earlier gene expression profiling studies have suggested that *trophinin* or *MAGE-D3* transcript could be detected in mouse brain, testis, and kidney (also in thymus, parathyroid gland, ovary, prostate, epididymus, and embryo), but not in the rest of the organs [16, 21]. In agreement with these reports, flow cytometric analysis with Kiatomab showed that KIAA1114 is expressed on the surface of cells of testis and kidney, but not by the cells of spleen and lung (Figure 1D), suggesting that surface binding of Kiatomab is specific to cells that express *trophinin* mRNA. KIAA1114 expression in these organs was also validated by Western blot analysis, showing the presence of KIAA1114-specific bands in testis and kidney, but not in spleen and lung.

Overall, these results not only provide the first empirical evidence that KIAA1114 is indeed expressed at the protein level, but also propose that it is localized mainly on the cell surface.

### KIAA1114 is overexpressed in human liver cancer cell lines with aggressive characteristics and HCC patient's primary tumor

Previous studies have suggested that the expression of TROPHININ was frequently observed in patients bearing malignant or metastatic tumors, including testicular germ tumor and adenocarcinomas of colon and lung [12]. To investigate whether KIAA1114 shows analogous pattern of expression in cancer, epithelial cell lines of various tissue origin were subjected to flow cytometric analysis. Kiatomab showed reactivity towards the majority of tumor cell lines, although the degree of KIAA1114 expression varied among different cell lines, even within those from the same tumor type (Supplementary Table 1).

**Figure 2:**
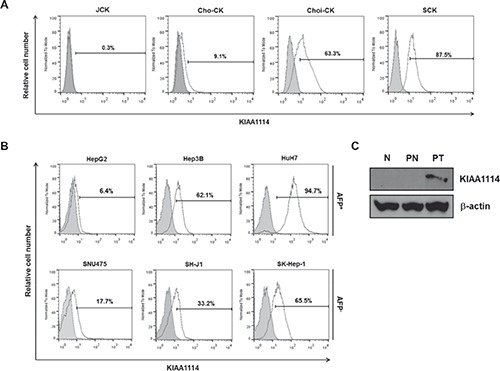
KIAA1114 expression in human liver cancer cell lines and primary tumors (**A** and **B**) Flow cytometric analysis of surface KIAA1114 expression in human CC cell lines **(A)** and AFP^+^ and AFP^−^ HCC cell lines **(B)**. **(C)** Immunoblot analysis of tissue lysates from normal liver (N), patient tumor (PT), and its adjacent normal tissue from the same donor (PN). β-actin served as an internal control.

Among the various types of tumors screened, we focused on exploring the role of KIAA1114 in human liver cancer, including HCC and cholangiocarcinoma (CC), based on our observation of the potential correlation between KIAA1114 expression levels and tumor progression. Firstly, in CC, KIAA1114 expression was significantly higher in sarcomatoid SCK cells than adenocarcinomatous cell lines, JCK, Cho-CK, and Choi-CK (Figure 2A). A prior study showed that SCK cells not only possessed strong resistance to chemotherapeutic agents, but also expressed elevated levels of genes involved in cancer progression and metastasis as well as EMT-associated markers [23, 24]. As sarcomatoid CCs are known to be more aggressive and have worse prognosis than adenocarcinomatous CCs [24], increased expression of KIAA1114 may contribute to the acquisition of an aggressive phenotype in CC. It was also worth noting that, among three adenocarcinomatous cell lines, Choi-CK cell line established from a stage IVB patient exhibited much higher KIAA1114 expression than did Cho-CK and JCK cells, both of which were derived from stage IVA patients [24].

The possible involvement of KIAA1114 in tumor progression was more thoroughly investigated in HCC. Among three AFP-producing HCC cell lines, HepG2 cells showed the lowest level of KIAA1114 expression, whereas HuH7 cells displayed the highest expression level (Figure 2B). Based on previous studies that characterized in vivo tumorigenicity of different HCC cell lines (tumorigenicity in the order of HuH7 > Hep3B > HepG2) [25], we found that KIAA1114 expression correlates with tumorigenic potential. Similar results were observed in AFP^−^ cell lines where highly tumorigenic SK-Hep-1 cells displayed greater KIAA1114 expression than non-tumorigenic SNU475 cells.

To assess the clinical relevance of KIAA1114 to human liver cancer, tissues lysates obtained from primary HCC, matched non-tumor, and normal liver were analyzed by Western blot for KIAA1114 expression. Kiatomab failed to detect the band that corresponds to KIAA1114 in normal liver tissue from a healthy donor. This result is consistent with a previous study that showed the absence of *trophinin* (or *MAGE-D3*)-specific signal in the adult liver of mice by semiquantitative RNA dot blot analysis [16]. More importantly, KIAA1114 expression was solely detected in the primary liver tumor, but not in the adjacent non-malignant hepatic tissue derived from the same patient (Figure 2C).

### KIAA1114^high^ cells isolated from HCC cell lines possess TIC-like properties

**Figure 3:**
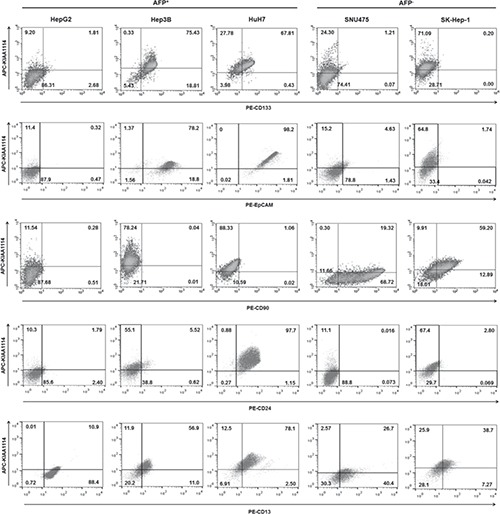
Overlapping expression between established liver TIC markers and KIAA1114 Flow cytometric analysis of HCC cell lines stained with antibodies against CD133, EpCAM, CD90, CD24, and CD13 in conjunction with Kiatomab. The quadrant marker of each plot was set on the basis of respective isotype control staining.

Positive correlation between tumorigenicity of HCC cell lines and expression level of TIC markers has been observed in earlier studies. For example, the expression of CD133, the first TIC marker identified in liver cancer, was markedly higher in HCC cell lines having tumor-initiating capabilities in vivo compared to those lacking such capacities [26]. Similar results were obtained with another TIC marker CD90, whose expression level was closely associated with tumorigenic as well as metastatic potential of liver cancer cell lines [25]. Based on these results, we hypothesized that KIAA1114 may also be used as a marker to isolate TIC population. To validate this, we first determined whether the expression pattern of KIAA1114 overlapped with those of known liver TIC markers in HCC cell lines (Figure 3). Differential expression of various TIC markers between AFP^+^ and AFP^−^ cells was also evaluated, as previous studies have shown that distinct prognostic subtypes of HCCs displayed distinguishable levels of expression for stem/maturation markers, including CD133 and CD90 (Supplementary Table 2) [8]. Firstly, the expression of KIAA1114 clearly overlapped with that of CD133 and EpCAM, both of which were expressed in AFP^+^ HCC cell lines. The level of CD133 and EpCAM expression was associated with tumorigenic potentials of AFP^+^ cell lines in general, as more tumorigenic Hep3B and HuH7 cells displayed significantly higher expression of those markers while less tumorigenic HepG2 cells showed no apparent expression. Nonetheless, CD133 and EpCAM expressions did not distinguish HuH7 from Hep3B cells whose tumorigenic capacity was known to be inferior to the former. It was also important to note that both CD133 and EpCAM were not or minimally expressed by AFP^−^ HCC cell lines. Conversely, CD90 expression was confined to AFP^−^ cells, in which a fraction of CD90^+^ cells was co-localized with KIAA1114-expressing cells. Nevertheless, non-tumorigenic SNU475 cells expressed higher level of CD90 than highly tumorigenic SK-Hep-1 cells. CD24 is a recently discovered liver TIC marker whose expression was found to be upregulated in residual chemoresistant HCC xenograft tumors [27]. Although this previous study reported that CD24 was expressed in AFP^+^ cell lines, including Hep3B and HuH7, as well as in a AFP^−^ cell line, HLE, our results revealed that only AFP^+^ HuH7 cells expressed significantly high level of CD24 and displayed discernible co-localization of CD24^+^ and KIAA1114-expressing cells. Accordingly, a definite correlation between the extent of CD24 expression and tumorigenic potentials of cell lines was only observed in AFP^+^ subtype. Lastly, flow cytometric analysis of CD13, a semiquiescent TIC marker regulating ROS-induced DNA damage [28], showed that its expression could be detected and overlapped with that of KIAA1114 in all cell lines tested, regardless of their AFP expression status. Yet, CD13 expression showed no correlation with tumorigenicity of HCC cell lines in any of the HCC subtypes. These findings suggest that KIAA1114 is a unique biomarker whose expression not only overlaps with diverse liver TIC markers, but also correlates with tumorigenic capacities of HCC cell lines in both AFP^+^ and AFP^−^ subtypes.

**Figure 4:**
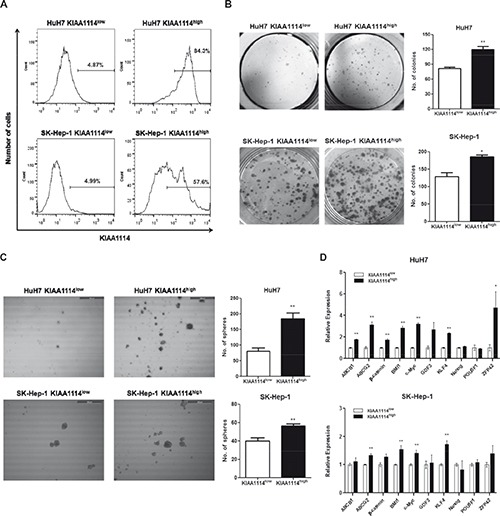

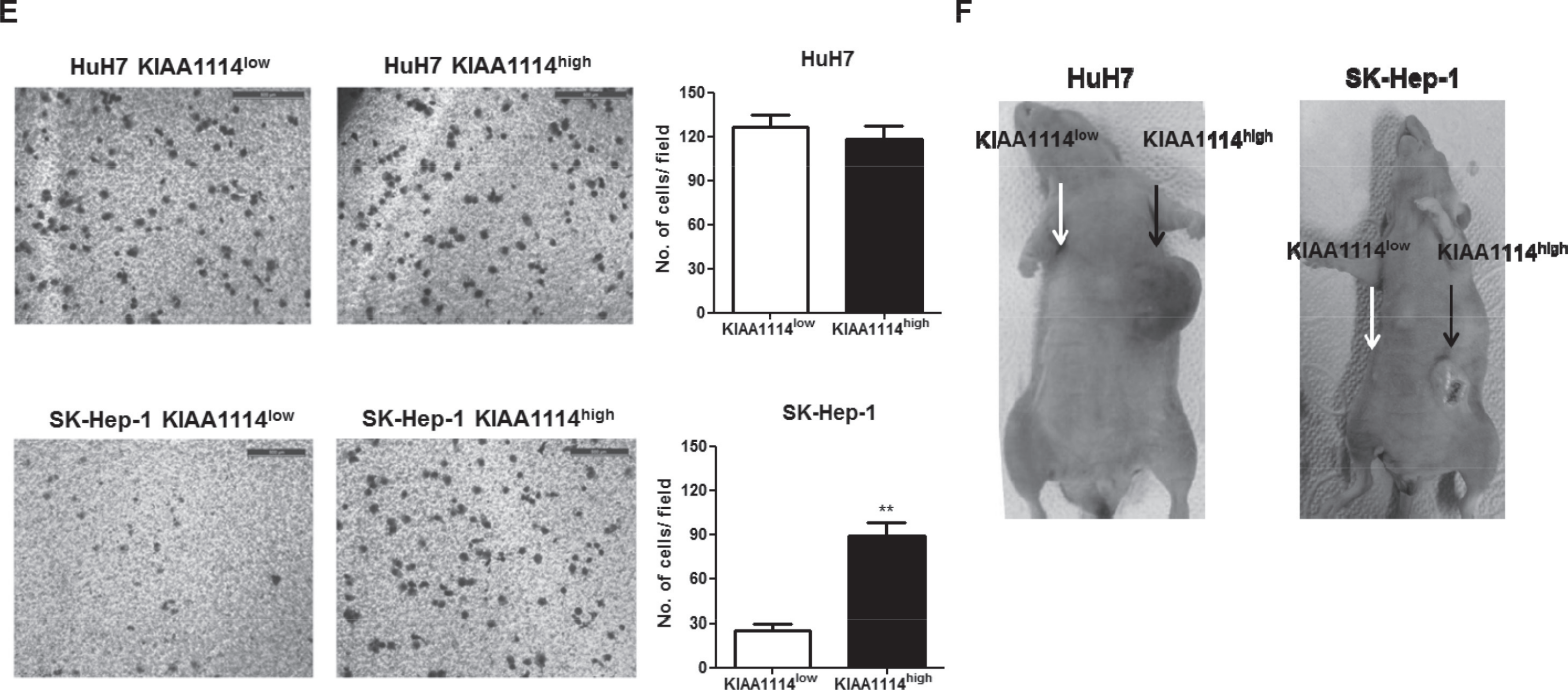
TIC-like properties of KIAA1114^high^ HCC cells **(A)** Flow cytometric analysis of sorted KIAA1114^high^ and KIAA1114^low^ cells from HuH7 and SK-Hep-1 cell lines. **(B)** Colony formation assay of KIAA1114^high^ and KIAA1114^low^ fractions isolated from HuH7 and SK-Hep-1 cells. 2,000 sorted cells were seeded into 6-well plates in triplicate and cultured for 2 weeks. The resulting colonies were stained with crystal violet and counted under the microscope. Representative images are shown. *p <0.05; **p<0.01. **(C)** Sphere forming assay of purified cells. 2,000 KIAA1114^high^ and KIAA1114^low^ cells were plated in 6-well ultra-low attachment dishes and cultured for 2 weeks. Spheres were counted and photographed using the microscope. Scale bars: 500 μm. **p <0.01. **(D)** Quantitative real-time PCR analysis of stemness-related genes in KIAA1114^high^ and KIAA1114^low^ cells derived from HuH7 and SK-Hep-1.*p <0.05; **p<0.01. **(E)** Transwell migration assay of KIAA1114^high^ and KIAA1114^low^ subsets. 5 × 10^4^ HuH7-sorted or 1 × 10^4^ SK-Hep-1-sorted cells were seeded onto the upper chamber and incubated for 16 hours. Migrated cells were stained with Diff-Quik and counted. Representative microscopic fields are shown. Scale bars: 500 μm. **p <0.01. **(F)** Representative images of beige/nude/xid mice subcutaneously injected with 1 × 10^4^ HuH7-sorted or 1 × 10^5^ SK-Hep-1-sorted KIAA1114^high^ cells (black arrow) and KIAA1114^low^ cells (white arrow). The photographs were taken 3 months after injections. All graphically represented data are shown as average ± SEM.

Nextly, to investigate whether KIAA1114 could play a role as an independent marker to identify liver TICs, we isolated cell populations expressing high and low levels of KIAA1114 from AFP^+^ HuH7 cells and AFP^−^ SK-Hep-1 cells and comparatively assessed their functional capacities of TICs. As with previous studies of CD133 and EpCAM expression analysis in human liver cancer [8, 26], HCC cell lines examined in this study exhibited a Gaussian distribution of KIAA1114 expression levels, rather than displaying a discrete, isolated population of KIAA1114^+^ cells. Therefore, sorted fractions obtained by FACS-selecting the top 10% most brightly stained cells were designated “KIAA1114^high^” cells, whereas those derived from the bottom 10% most dimly stained cells were designated “KIAA1114^low^” cells. Assuming 95% purities of KIAA1114^low^ cells after separation, the purities of KIAA1114^high^ cells in HuH7 and SK-Hep-1 cells were 84.3% and 57.6%, respectively (Figure 4A). Nextly, to evaluate clonogenic potential of isolated subpopulations, colony formation assay was performed. The result showed that the number of colonies generated from KIAA1114^high^ populations was greater than that from KIAA1114^low^ counterparts, regardless of AFP expression status of parental cells (Figure 4B). Analogously, sphere-formation assay, which was used as readout for self-renewal activity, revealed that KIAA1114^high^ cells isolated from either HuH7 or SK-Hep-1 cell line formed significantly more spheroids than did KIAA1114^low^ cells (Figure 4C). These findings suggest that KIAA1114^high^ subsets possess higher proliferative potential and self-renewal capacity than KIAA1114^low^ counterparts. We also noticed differences in qualitative aspects of spheres formed by AFP^+^ HuH7 and AFP^−^ SK-Hep-1 cells. The size of each spheroid generated by KIAA1114^high^ fraction was much larger than that of KIAA1114^low^ fraction-derived one in HuH7 cells, while the spheres formed by KIAA1114^high^ and KIAA1114^low^ cells sorted from SK-Hep-1 cells were similar in size. Moreover, the overall sphere-forming efficiencies of sorted cells were relatively lower in SK-Hep-1 cells compared to HuH7 cells.

The assessment of differentiating capacity of isolated tumor cells is another criterion for evaluating TICs, as these cells are known to undergo asymmetric divisions to generate heterogeneous populations containing phenotypically diverse cell types [2]. Hence, we analyzed cell surface KIAA1114 expression on short-term passages of KIAA1114^high^ and KIAA1114^low^ subpopulations sorted from HuH7 and SK-Hep-1 cell lines by flow cytometry to analyze whether KIAA1114^high^ HCC cells could produce KIAA1114^low^ cells and vice versa. In both AFP^+^ and AFP^−^ HCC subtypes, KIAA1114^high^ cells generated increasing numbers of KIAA1114^low^ cells until KIAA1114 expression reached steady-state levels (day 15), as shown by time-dependent decreases in the fluorescence intensities, suggesting the differentiation of KIAA1114^high^ cells into KIAA1114^low^ counterparts (Supplementary Figure 2). Unexpectedly, we also observed gradual upregulation of KIAA1114 expression in KIAA1114^low^ fractions, yielding contradictory results from previous reports of EpCAM and CD24 where EpCAM^−^ and CD24^−^ HCC fractions maintained their low marker expression status for 2 weeks in culture, respectively [8, 27]. However, our results are in line with a prior study of CD133 that showed increased CD133 surface expression upon culture in CD133^−^ subsets isolated from HCC cell lines as well as xenograft tumors. These data were interpreted as the consequence of asymmetric division undergone by a small subset (~5%) of CD133^+^ cells contaminated in the CD133^−^ population [26]. Therefore, considering the moderate purity of sorted HCC cells used in the present study (Figure 4A), it could be presumed that KIAA1114^low^ subsets contain contaminating KIAA1114^high^ cells abundant enough to generate both KIAA1114^high^ and KIAA1114^low^ populations.

Previous studies have shown that the majority of TICs, including those identified in HCC, exhibited elevated expression levels of “stemness” genes, which are defined as genes required for stem cell maintenance by conferring pluripotency, compared to their differentiated counterparts [29, 30]. In addition, TICs often display upregulated expression of ABC superfamily members that render them resistant to various types of therapies. Therefore, we assessed the expression levels of 8 stemness-associated genes (*β-catenin, BMI-1, c-Myc, GDF3, KLF4, Nanog, POU5F1,* and *ZFP42*) and 2 ABC transporter genes (*ABCB1* and *ABCG2*) in KIAA1114^high^ and KIAA1114^low^ cells (Figure 4D). In HuH7 cells, 5 stemness-related genes (*β-catenin, BMI1, c-Myc, KLF4,* and *ZFP42*) and all of ABC transporter genes were upregulated in KIAA1114^high^ fraction in a statistically significant manner. Among them, the expression levels of *ABCG2*, *c-Myc*, and *ZFP42* were 3- to 5-fold higher in KIAA1114^high^ cells compared to KIAA1114^low^ cells. Contrarily, the overexpression of stemness-associated genes was not clearly observed in AFP^−^ SK-Hep-1 cell line with only 4 genes (*ABCG2*, *BMI*, *c-Myc*, and *KLF4*) showing marginal upregulation in KIAA1114^high^ cells compared to KIAA1114^low^ cells. Since AFP^−^ HCC cell lines are reported to express genes related to EMT and mesenchymal lineages rather than hepatic stem cell-specific markers [9], sorted KIAA1114^high^ and KIAA1114^low^ SK-Hep-1 cells were then subjected to transwell migration assay, a common method for analyzing post-EMT activity [31]. Intriguingly, KIAA1114^high^ fractions showed more than 3-fold higher migration rate compared to KIAA1114^low^ counterpart. On the other hand, KIAA1114^high^ and KIAA1114^low^ cells isolated from HuH7 cells displayed comparable migration capacities (Figure 4E). These results suggest that KIAA1114^high^ cells derived from SK-Hep-1 cells may retain discrete characteristics and gene expression profiles compared with those from HuH7 cells.

As mentioned earlier, the definition of TICs involves empirical evaluation of functional qualities of a given cell population, especially in its capacity to initiate and sustain tumor growth in immunodeficient mice. Accordingly, we assessed in vivo tumorigenic capacities of KIAA1114^high^ and KIAA1114^low^ cells by subcutaneously injecting them into beige/nude/xid mice. For HuH7 cell line, as few as 1,000 KIAA1114^high^ cells were sufficient to establish tumors in 100% of mice, while the same number of KIAA1114^low^ cells was unable to develop tumors in any of the mice injected. As many as 100,000 KIAA1114^low^ cells were required to accomplish consistent tumor generation, indicating about 100-fold lower tumor-forming efficiency compared to KIAA1114^high^ counterpart. In the case of SK-Hep-1 cell line, isolated KIAA1114^high^ fraction not only developed tumors in 2 out of 4 mice with 10,000 cells, but also achieved 100% tumor incidence with 100,000 cells. On the contrary, KIAA1114^low^ fraction failed to initiate tumors in any mice, even with the highest cell number examined (100,000 cells/site) (Table 1). Notably, 10,000 HuH7-sorted KIAA1114^high^ cells formed much larger tumors than did 100,000 SK-Hep-1-isolated KIAA1114^high^ cells in the same amount of time (Figure 4F). This result is consistent with a previous study that EpCAM^+^ cells derived from AFP^+^ HCCs generated much larger and faster-growing tumors than did CD90^+^ cells purified from AFP^−^ HCCs [9].

**Table 1:** In vivo tumorigenicity of KIAA1114^high^ and KIAA1114^low^ cells isolated from HuH7 and SK-Hep-1 cell lines

Cell type	Cell numbers injected	No. of mice with tumors / Total No. of mice injected		
		2 Months	3 months	4 months
HuH7 KIAA1114^high^	1,000	0/4	2/4	2/2
	10,000	2/4	2/2	
	100,000	4/4		
HuH7 KIAA1114^low^	1,000	0/4	0/4	0/4
	10,000	0/4	0/4	1/4
	100,000	1/4	2/3	1/1
SK-Hep-1 KIAA1114^high^	1,000	0/4	0/4	0/4
	10,000	0/4	1/4	1/3
	100,000	3/4	1/1	
SK-Hep-1 KIAA1114^low^	1,000	0/4	0/4	0/4
	10,000	0/4	0/4	0/4
	100,000	0/4	0/4	0/4

## DISCUSSION

Cancer stem cells (CSCs), a tumorigenic subpopulation of cancer cells that drives unlimited propagation of malignant clones, are regarded as the major source of tumor recurrence and metastasis [32]. Accordingly, the identification and functional characterization of CSC-specific markers and phenotypic analysis of these marker-expressing cells are crucial for developing CSC-directed therapies. In the present study, we proposed the role of a novel tumor-associated antigen, KIAA1114, as a bona fide marker for liver CSCs, based on the functional and phenotypic features displayed by isolated KIAA1114^high^ HCCs in several CSC assays. Firstly, anchorage-dependent colony formation assay revealed that KIAA1114^high^ cells derived from both AFP^+^ and AFP^−^ cell lines retained superior proliferative capacities than KIAA1114^low^ cells. These results are in line with previous reports that showed enhanced colony-forming abilities of CD133^+^ and EpCAM^+^ HCC cells compared to their respective CD133^−^ and EpCAM^−^ counterparts, but contrast with the studies that displayed slower growth rate of CD13^+^ and CD24^+^ sorted cells than corresponding marker-negative fractions [8, 26-28]. These findings suggest that KIAA1114^high^ cells do not possess quiescent properties, which are occasionally ascribed to CSCs [2], as do CD13^+^ and CD24^+^ HCCs. Secondly, non-adherent sphere assay revealed that KIAA1114^high^ subsets exhibited stronger self-renewal capacities than KIAA1114^low^ subsets in both AFP^+^ and AFP^−^ HCCs. Analogous results were obtained in a prior study, where CD133^+^ and EpCAM^+^ cells isolated from HuH7 cell line exhibited higher spheroid-forming efficiencies than CD133^−^ and EpCAM^−^ cells, respectively [33]. Thirdly, and most importantly, in vivo limiting dilution assay provided direct evidence that KIAA1114^high^ fractions sorted from HCC cell lines had superior tumor-initiating capacities compared to KIAA1114^low^ cells. Since the operational definition of CSC was developed based on the capacity of cells to initiate and maintain tumor growth in immunodeficient mice [1], it might be feasible to designate KIAA1114^high^ HCCs as CSCs, or more accurately, TICs. Collectively, our findings suggest that KIAA1114^high^ HCCs recapitulate some of the basic but essential features of existing liver TICs, including enhanced capacities for proliferation, self-renewal, and *in vivo* tumor generation, as those exhibited by CD133^+^ and EpCAM^+^ cells.

The reliability of existing liver TIC markers has been frequently challenged based on the heterogeneous and polyclonal nature of HCC that distinct phenotypic markers are expressed by different histological subtypes [34]. Previous studies have shown that HCC can be classified into four subtypes based on EpCAM and AFP expression status and each subtype has different molecular signatures and discrete signaling pathways that regulate its biological activities [35]. These results led us to focus on the investigation of differential expression patterns of KIAA1114 and known liver TIC markers between AFP^+^ and AFP^−^ subtypes. Consistent with previous studies, our results showed that AFP^+^ cell lines expressed CD133 and EpCAM, while AFP^−^ cells only expressed CD90 [8]. On the other hand, KIAA1114 expression was detected in all cell lines tested and isolated KIAA1114^high^ cells exhibited much stronger tumorigenic potential than did KIAA1114^low^ cells in both HCC subtypes. Interestingly, however, stemness-associated gene expression profiles and migratory properties of KIAA1114^high^ subsets were shown to be different in AFP^+^ HuH7 cells and AFP^−^ SK-Hep-1 cells. HuH7-isolated KIAA1114^high^ cells exhibited tendency to express significantly higher levels of stemness genes while maintaining equivalent migration capacities compared to KIAA1114^low^ cells. In contrast, SK-Hep-1 sorted KIAA1114^high^ cells displayed markedly enhanced migration without the upregulation of stemness-related markers. Such discrepancies in the nature of TICs isolated from distinct HCC subtypes is well-characterized in a recent study, which showed that EpCAM^+^ cells isolated from AFP^+^ cell lines exerted highly tumorigenic properties in vivo and expressed hepatic stem cell-associated genes while lacking capacities to metastasize [9]. Conversely, CD90^+^ cells was unable to produce large subcutaneous tumors but capable of forming lung metastases with upregulated expression of mesenchymal lineage markers. Although genomic profiles of KIAA1114^high^ and KIAA1114^low^ cells were not extensively investigated in the current study, it is reasonable to predict that KIAA1114^high^ cells purified from SK-Hep-1 cells might have increased expression of genes associated with metastatic progression or EMT. Aside from KIAA1114, CD13 was the only marker to be expressed in both AFP^+^ and AFP^−^ cell lines, but it remains to be determined whether CD13 could mark tumorigenic populations in AFP^−^ HCCs. To our knowledge, this is the first experimental study to isolate tumorigenic, liver TIC-like populations from both AFP^+^ and AFP^−^ HCC subtypes utilizing a single biomarker.

A potential function of MAGE family members as a marker for (cancer) stem cells has been initially proposed by Simpson et al. that cancer-testis antigens encoded by genes located on X chromosome, including *MAGE-A*, *-B*, *-C*, and *-D*, are frequently expressed in the spermatogonia, and therefore serve a putative role in marking male germ line stem cells [36]. Definitive experimental evidence for the association of MAGE proteins with stem cell phenotype was later provided by expression profiling studies of various types of human stem cells. For instance, MAGE-A and N-RAGE (also known as MAGE-D1) were expressed by mesenchymal stem cells, but their expression levels decreased upon the differentiation of stem cells [37]. Similarly, the expression of MAGE-A3 and -A6 were detected in embryonic stem cells, but not in differentiated derivatives [38]. In addition, global gene expression analysis of CD133^+^ cells isolated from four different cord blood units identified *trophinin* as one of 32 genes that encode membrane proteins selectively expressed on CD133^+^ hematopoietic stem cells [39]. Although specific functions and mechanisms of these MAGE proteins in stem cell biology remain largely unknown, all of the above-mentioned studies implicate the potential role of MAGE family members in the regulation of stem cell maintenance or differentiation. More importantly, gene expression analysis of 31 different cancer-testis antigens in tumor spheres derived from 4 human glioma cell lines and a primary tumor specimen revealed that *MAGE-D3* (which is equivalent to *trophinin*) was the only gene that showed increased expression in all examined tumor spheres compared to their differentiated counterparts [40]. Since sphere-forming cells are known to exhibit higher self-renewal capacity, proliferative potential, drug resistance, and in vivo tumorigenic/metastatic capabilities than parental tumor cells [32, 41], *trophinin* gene products including KIAA1114 may be involved in promoting one or more of these biological processes in glioma model as well. Therefore, these findings raise the possibility that KIAA1114 may not only play a role as an alternate marker of hematopoietic stem cells, but also act as a broad-spectrum TIC marker for various types of tumors, including HCC and glioma.

In summary, our study characterized KIAA1114 as a novel cell-surface marker of liver TICs using human HCC cell lines and showed that isolated KIAA1114^high^ fractions from two different HCC subsets (AFP^+^ and AFP^−^) displayed subtype-specific TIC features, resembling those of EpCAM^+^ TICs in AFP^+^ HCC and CD90^+^ TICs in AFP^−^ HCC. Although the applicability of KIAA1114 as a single biomarker to isolate TICs from multiple HCC subtypes offers unique advantages over other established TIC markers, it is crucial to validate its function by sorting KIAA1114^high^ cells from clinical samples. We could not resolve this issue due to stringent regulatory restrictions on the acquisition of human primary solid tumor specimens imposed by institutional review boards in Korean domestic hospitals. However, substantial evidence has been provided by a number of studies that many known liver TIC markers verified in cell lines were also applicable to primary HCC. For instance, a previous study has shown that functional CD90^+^ cells could be isolated from 13 out of 28 HCC specimens and all of them could initiate tumor growth in immunodeficient mice with ≤10,000 cells within four months [25]. Similarly, EpCAM^+^ and CD24^+^ cells derived from patient samples displayed superior tumorigenic capabilities *in vivo* than did EpCAM^−^ and CD24^−^counterparts, respectively [8, 27]. Considering the overlapping pattern of expression between existing liver TIC markers and KIAA1114 and the resemblance in the functional features of isolated KIAA1114^high^ cells to those of EpCAM^+^ and CD90^+^ cells, it is tempting to speculate that KIAA1114 could serve a parallel role in patient-derived tumors as a TIC marker.

## MATERIALS AND METHODS

### Anti-human KIAA1114 mAb generation

Murine hybridoma cell line producing anti-KIAA1114 mAb was kindly provided by Genexine Co., Ltd.. Briefly, Balb/c mice were immunized with a gene encoding N-terminal region (amino acids 1-366) of human KIAA1114. Splenocytes were collected from immunized mice and fused with SP2/0 myeloma cells. The hybridoma clone secreting mAb with the strongest antigen binding activity was selected and further characterized. mAb derived from selected clone was designated Kiatomab (IgG2b, κ). For the production of Kiatomab, Balb/c mice were first intraperitoneally treated with 500 μl Pristane (Sigma Aldrich). After 7 days, 5 X 10^6^ cells of hybridoma were injected intraperitoneally and the ascitic fluid was collected when abdominal bloating was observed. The purification of mAb was conducted with the AKTA purifier system using HiTrap Protein G HP column (GE Healthcare).

### Cell lines

Human CC cell lines and a HCC cell line, SH-J1, were kindly provided by Dr. Dae-Ghon Kim (Chonbuk National University Medical School, Korea) and grown in DMEM (WelGENE). Other HCC cell lines were generously provided by Dr. Kwan Yong Choi (POSTECH, Korea) and grown in RPMI1640 (WelGENE). Human embryonic kidney 293T cell line was purchased from American Type Culture Collection and maintained in recommended medium. All medium was supplemented with 10% FBS (Thermo Scientific) and antibiotic-antimycotic (Invitrogen).

### Mice

Balb/c mice were purchased from Charles River Breeding Laboratories. Beige/nude/XID mice were purchased from The Jackson Laboratory. Animals were maintained under specific pathogen-free conditions. All animal procedures were approved by the Animal Care Committee of POSTECH Biotech Center.

### Western Blotting

Cell lysis was performed as previously described [42]. Whole cell lysates were incubated in 37°C for 30 minutes, resolved in 4–15% Mini-PROTEIN TGX Precast Gel (Bio-Rad), and transferred to nitrocellulose membrane (Whatman). Membranes were blocked 1 hour with 5% non fat dry milk in 0.1% Tween TBS (TBST) and further incubated overnight at 4°C with Kiatomab or anti-TROPHININ mAb (clone 3–11) (Abcam) diluted in blocking buffer. After washing with TBST, membranes were incubated at room temperature for 1 hour with horseradish peroxidase (HRP)-conjugated anti-mouse IgG (Bio-rad). Immunoreactive bands were stained with Super Signal West Pico Chemiluminescent kit (Pierce) and exposed to X-ray film. For Western blot analysis of extracted proteins from tissue, mouse brain tissues were minced and homogenized in 3ml RIPA buffer per gram of tissue. After centrifugation, supernatant containing the total cell lysate was removed and subjected to SDS-PAGE. Human liver whole tissue lysate and paired primary tumor and normal tissues were purchased from Novus Biologicals.

### Flow cytometry

Single cell suspensions from diverse murine tissues were prepared as previously described [43]. Cells were resuspended in FACS buffer (PBS/1% FBS) containing Kiatomab or purified mouse IgG2b isotype antibody (eBioscience) and incubated on ice for 15 minutes. After being washed with FACS buffer, cells were incubated with allophycocyanin (APC)-conjugated goat anti-mouse Ig (BD Biosciences). For double fluorescence staining, cells were additionally stained with phycoerythrin (PE)-conjugated CD133 (MiltenyiBiotec), CD90, EpCAM, CD24, or CD13 (eBioscience). Single cell sorting was performed with MoFlo XDP cell sorter (Beckman Coulter) by gating the top 10% of the most brightly fluorescent cells and the bottom 10% of the most dimly fluorescent cells.

### Colony and sphere formation assay

Colony- and sphere-formation assays were performed as previously described [26, 44]. Cells were seeded at a density of 2000 cells per well and the resulting colonies or spheres were counted under the microscope at day 14.

### Stemness gene expression analysis

RNA extraction and cDNA synthesis from isolated KIAA1114^high^ and KIAA1114^low^ cells were performed using Total RNA Extraction Kit (Intron Biotechnology) and QuantiTect Reverse Transcription Kit (Qiagen), respectively. Real-time PCR was performed on a Viaa 7 real-time PCR system (Applied Biosystems) with various stemness gene-specific primers (Supplementary Table 3) and SYBR Green PCR Master Mix (Applied Biosystems).

### Transwell migration assay

5 X 10**^4^** cells of HuH7-sorted or 1 X 10**^4^** cells of SK-Hep-1 sorted fractions were resuspended in serum-free medium and placed into the upper chambers of 24-well transwell (8 μm pore size; Corning). The lower chambers were filled with the medium containing 10% FBS. After incubating transwells at 37°C for 16 hours, migrated cells were stained using Diff-Quik Stain Set (Dade Behring, Inc.) and counted in 16 random fields under the microscope.

### In vivo tumorigenicity assay

Beige/nude/XID mice were subcutaneously injected with various numbers of sorted KIAA1114^high^ and KIAA1114^low^ cells. Mice were monitored up to 4 months for the formation of palpable tumors.

### Statistics

Data were expressed as mean ± SEM. Statistical differences between groups were calculated by a 2-tailed Student t test.

## SUPPLEMENTARY FIGURES AND TABLES


