# Supplementary material for: KIAA1114, a full-length protein encoded by the trophinin gene, is a novel surface marker for isolating tumor-initiating cells of multiple hepatocellular carcinoma subtypes
Source: Oncotarget. 2014 Mar 31;5(5):1226–40. doi: 10.18632/oncotarget.1677 (PMC4012722; doi:10.18632/oncotarget.1677)
Supplement: Supplementary file 1 [file oncotarget-05-1226-s001.pdf]

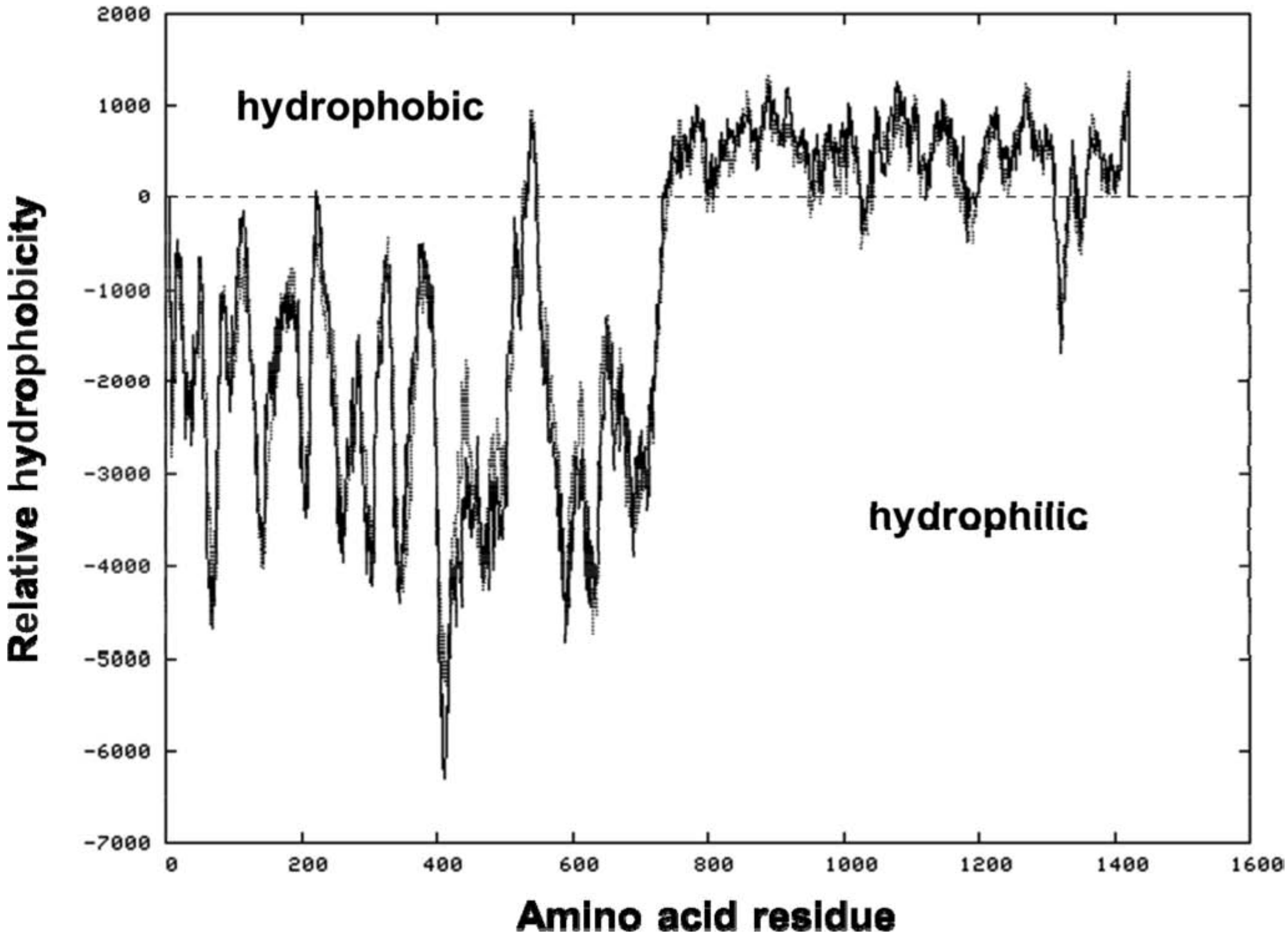

Supplementary Figure S1: Hydropathy analysis of human KIAA1114. The hydropathy plot of human KIAA1114 was generated by TMPred program (http://www.ch.embnet.org/software/TMPRED_form.html).

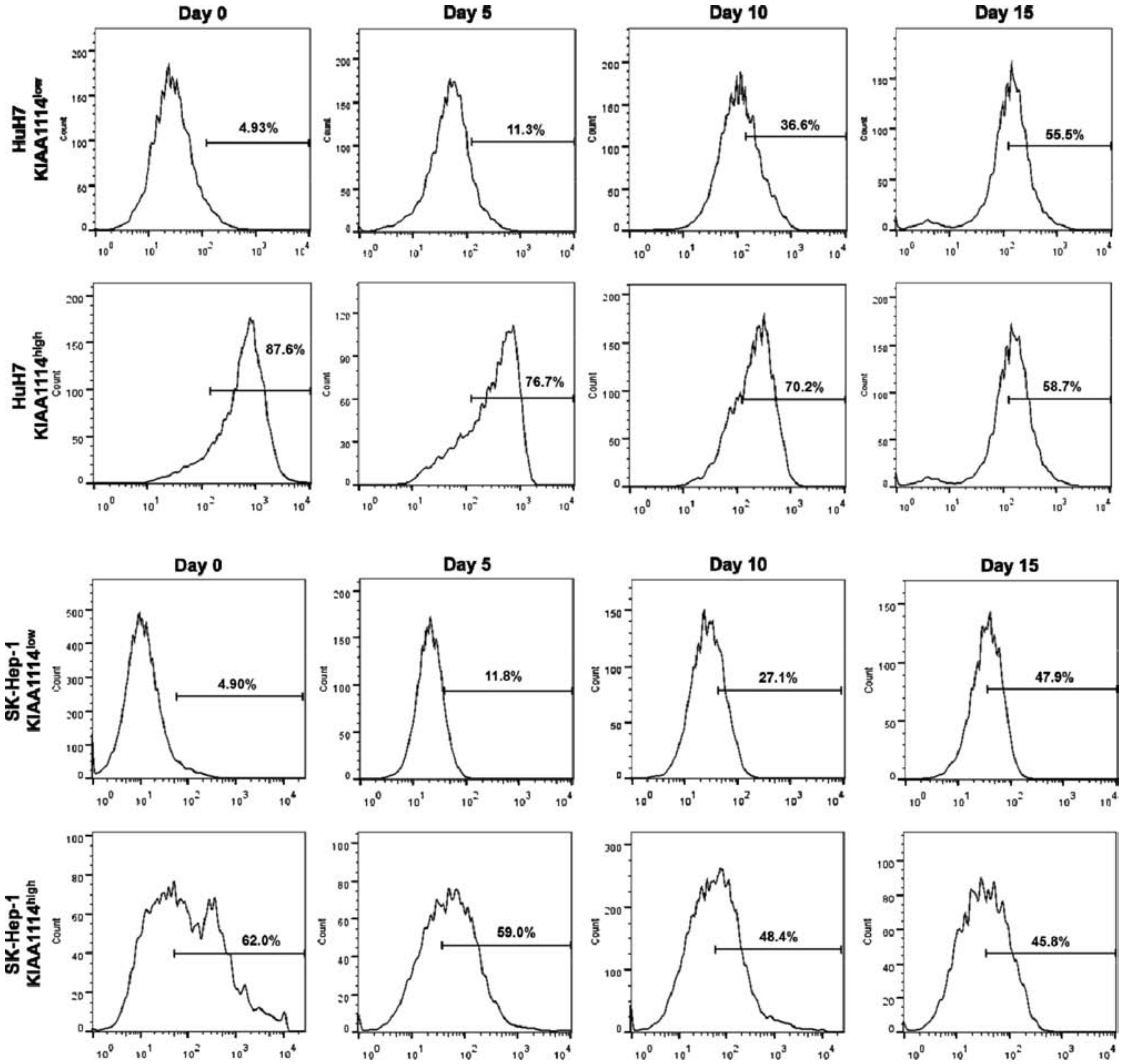

Supplementary Figure S2: KIAA1114 expression in sorted HCC fractions upon short-term culture. Flow cytometric analysis of KIAA1114 expression in HuH7- and SK-Hep-1 derived KIAA1114^{high} and KIAA1114^{low} cells immediately after isolation and after culturing for 5, 10, and 15 days.

Supplementary Table S1: KIAA1114 expression in epithelial cancer cell lines

Liver (HCC)			Liver (CC)	
**Cell line (origin)**	**KIAA1114 (%)[a]**		**Cell line (origin)**	**KIAA1114 (%)[a]**
HepG2 (H)	10.5±4.2		JCK (H)	0.5±0.2
Hep3B (H)	71.6±7.1		Cho-CK (H)	8.3±2.2
HuH7 (H)	94.5±3.8		Choi-CK (H)	62.8±5.5
SNU475 (H)	21.1±6.2		SCK (H)	88±4.3
SH-J1 (H)	27.8±5.5			
SK-Hep-1 (H)	68.3±2.5			

Breast			Brain, nervous system	
**Cell line (origin)**	**KIAA1114 (%)**		**Cell line (origin)**	**KIAA1114 (%)**
T47D (H)	44.1		U-373 MG (H)	31.6
MDA-MB-436 (H)	69.2		U-87 MG (H)	33.7
MDA-MB-468 (H)	70.8		U-343 MG (H)	37.8
SKBR3 (H)	74		U-118 MG (H)	57.4
MDA-MB-231 (H)	86.5		LN-18 (H)	74.6
4T1 (M)	94.2		GL-261 (M)	22.5

Colorectal			Ovary	
**Cell line (origin)**	**KIAA1114 (%)**		**Cell line (origin)**	**KIAA1114 (%)**
SW480 (H)	1.8		C13 (H)	8.2
LoVo (H)	37.5		OV2008 (H)	75.7
HCT-8 (H)	62.6		A2780-Tax[b] (H)	27.2
HCT116 (H)	97.9		A2780 (H)	81.4
MC38 (M)	63.3		SKOV3-Tax[b] (H)	19.8
CT26 (M)	97.2		SKOV3 (H)	64.3

Melanoma of skin			Kidney	
**Cell line (origin)**	**KIAA1114 (%)**		**Cell line (origin)**	**KIAA1114 (%)**
A375 (H)	15.5		A498 (H)	55.6
A375P (H)	18.5		786-O (H)	78.3
A375SM (H)	19.9		Renca (M)	20
B16F10 (M)	34.2			

[a] The percentage of KIAA1114^{high} cells in liver cancer cell lines were represented as mean ± SEM of eight independent experiments. KIAA1114 expression levels in other tissue-derived cell lines were represented as the mean percentage of at least three independent flow cytometric analyses.
[b] Taxol-resistant cell lines. H: Human origin; M: Mouse origin.

Supplementary Table S2: Expression levels of KIAA1114 and liver TIC markers in human HCC cell lines

Cell line	AFP	CD133 (%)	CD90 (%)	EpCAM (%)	CD13 (%)	CD24 (%)	KIAA1114 (%)
**HepG2**	+	4.7±1.2	0.5±0.3	0.9±0.2	99.2±4.7	3.3±1.1	10.5±4.2
**Hep3B**	+	93.2±3.7	0.04±0.01	97.6±3.7	63.4±7.4	7.6±1.8	70.6±9.1
**HuH7**	+	68.3±2.5	0.8±0.3	95.4±4.6	82.1±4.3	97.9±2.0	94.0±4.8
**SNU475**	−	0.9±0.4	88.1±3.2	5.2±2.3	63.2±6.2	0.07±0.02	19.1±8.2
**SH-J1**	−	0.2±0.05	97.5±4.6	0.6±0.09	99.0±3.8	0.1±0.03	34.7±6.1
**SK-Hep-1**	−	0.3±0.1	70.4±6.3	1.3±0.5	46.3±8.1	2.0±0.9	67.9±3.5

The numbers represent the mean ± SEM of six independent experiments.

Supplementary Table S3: Sequences of the primers used in the present study

Gene	Forward primer (5'-3')	Reverse primer (5'-3')
Stemness-related genes		
ABCB1	TCACTTCAGGAAGCAACCAG	ATTCCTCGAGAAACTGCGAA
ABCG2	CTGAGATCCTGAGCCTTTGG	AAGCCATTGGTGTTTCCTTG
β-catenin	AGGTCTGAGGAGCAGCTTCA	ATTGTCCACGCTGGATTTTC
BMI	AATCCCCACCTGATGTGTGT	GCTGGTCTCCAGGTAACGAA
c-Myc	GCTGCTTAGACGCTGGATTT	CACCGAGTCGTAGTCGAGGT
GDF3	TGCTACGTAAAGGAGCTGGG	TTCCCTTTCTTTGATGGCAG
KLF4	AGAGTTCCCATCTCAAGGCA	GTCAGTTCATCTGAGCGGG
Nanog	GATTTGTGGGCCTGAAGAAA	TTGGGACTGGTGGAAGAATC
POU5F1	ACTGCAGCAGATCAGCCACATCG	ATCCTCTCGTTGTGCATAGTCGC
ZFP42	GGTGGCATTGGAAATAGCAG	TGCCTAGTGTGCTGGTGGT
Housekeeping gene		
GADPH	AAGGTGAAGGTCGGAGTCAA	AATGAAGGGGTCATTGATGG
